# Elements of music that work to improve sleep, a narrative review

**DOI:** 10.3389/frsle.2025.1707162

**Published:** 2025-12-02

**Authors:** Ethan Y. Pan, Wei Wang

**Affiliations:** 1 Department of Radiology and Biomedical Imaging, University of California, San Francisco, San Francisco, CA, United States; 2 Bay Area Sleep Medicine, San Jose, CA, United States

**Keywords:** sleep health, insomnia, sleep quality, music characteristics, music dosing, randomized controlled trials, brain wave music, binaural beats

## Abstract

Sleep health is essential for overall wellbeing; however, millions of people worldwide experience poor sleep quality due to insomnia, stress, or lifestyle-related disturbances. Pharmacological and behavioral treatments, while effective, remain limited by side effects, accessibility barriers, or patient adherence. In contrast, music is an accessible, low-cost, and non-invasive intervention that is increasingly used by individuals to improve sleep. This narrative review synthesizes findings from randomized controlled trials and meta-analyses to identify the musical elements and delivery methods that are most effective in enhancing sleep quality. Across studies, listening to music consistently reduced sleep-onset latency, improved sleep efficiency, and increased total sleep time. Music that was slow in tempo (60–80 bpm), soft and smooth in melodies, instrumental, and simple in structure, often classical or new age, was most effective. Cultural familiarity, nature sounds, and religious music also demonstrated benefits in specific contexts. Innovative approaches, such as brain-wave music and binaural beats, show promise but require further validation. Optimal dosing included 30–45 min of daily listening before bedtime at comfortable volume levels. Despite strong evidence of short-term benefits, gaps remain in our understanding of the long-term effects, mechanisms of action, and impacts on youth populations. Future research should explore how personalized music interventions and artificial intelligence-generated compositions may advance sleep health. Overall, this review highlights the elements at work that make music a safe, scalable, and culturally adaptable adjunct to traditional sleep therapies.

## 1 Introduction

Healthy sleep is vital for physical and mental health and social wellbeing (Gothe et al., 2020; Grandner, 2022; Howarth and Miller, 2024; Moore et al., 2002). Sleep deficiency, including insufficient sleep, sleep disorders, and sleep disturbances, is widespread across different age and ethnic groups worldwide and is recognized as a global public health epidemic (Chattu et al., 2018; Lim et al., 2023). However, it is often overlooked, underreported, and carries significant economic costs. Sleep deficiency has been associated with a wide range of adverse health consequences, including cardiovascular disease, cancer, diabetes and other metabolic disorders, infection, hypertension, psychiatric and psychological disorders, traffic accidents, and occupational injuries (Chattu et al., 2018; Lim et al., 2023).

There are multiple treatment options available for common sleep disorders, such as insomnia, including maintaining good sleep hygiene, using medications, or engaging in psychological and behavioral therapies. Cognitive behavioral therapy for insomnia (CBT-I) is regarded as the first-line treatment for insomnia (Riemann et al., 2017). Despite its effectiveness, its application is restricted owing to the effort it demands and the limited availability of qualified professionals. Medications such as benzodiazepine receptor agonists are known to provide short-term relief and are widely used (Riemann et al., 2017). However, prolonged use of these drugs is linked to a higher risk of abuse, dependency, and side effects, such as lingering daytime drowsiness, cognitive issues, impaired motor skills, or even increased mortality (Frandsen et al., 2014; Jennum et al., 2015; Kripke et al., 2012). In addition to the limitations of current psychological and pharmacological treatments, many individuals with sleep problems do not seek professional help. Consequently, most people resort to non-medical self-help methods to enhance sleep quality, with listening to music being one of the most popular choices (Morin et al., 2006; Urponen et al., 1988). A simple online search for the terms “music” and “sleep” reveal a large market of music advertised for sleep-inducing or sleep-enhancing properties. For example, the most viewed YouTube video claiming to help with sleep has over 400 million views, highlighting a substantial interest in sleep-related music. However, the effectiveness of these commercial music products as a treatment for sleep disorders remains uncertain.

Music offers several benefits in enhancing sleep quality. It is easily accessible, cost-effective, and lacks the negative side effects associated with medications. Numerous studies have explored whether music can indeed improve sleep quality across different groups, and most of these studies have found that music enhances overall sleep quality and various sleep aspects, such as sleep latency, total sleep time, and sleep efficiency (the percentage of time spent asleep while in bed; de Niet et al., 2009; Feng et al., 2018; Jespersen et al., 2022). In a meta-analysis of 13 randomized controlled trials involving 1,007 participants, which focused on adults with insomnia listening to music (Jespersen et al., 2022), Jespersen et al. discovered moderate-certainty evidence that music groups experienced better sleep quality, as measured by the Pittsburgh Sleep Quality Index (PSQI), compared to those with no intervention or treatment as usual [TAU; mean difference (MD) −2.79, 95% confidence interval (CI) −3.86 to −1.72; 10 studies, 708 participants]. The PSQI scale ranges from 0 to 21, with higher scores indicating worse sleep. The effect size suggests an improvement in sleep quality equivalent to approximately one standard deviation favoring the intervention. The authors also found that, compared to no treatment or TAU, listening to music may alleviate issues with sleep-onset latency (MD −0.60, 95% CI −0.83 to −0.37; 3 studies, 197 participants), total sleep time (MD −0.69, 95% CI −1.16 to −0.23; 3 studies, 197 participants), and sleep efficiency (MD −0.96, 95% CI −1.38 to −0.54; 3 studies, 197 participants). In a meta-analysis conducted by Feng et al. in 2018, 20 randomized controlled trials were examined, involving 1,339 adult patients with primary insomnia and 12 different intervention arms (Feng et al., 2018). This study assessed the effectiveness of music listening compared to other interventions, such as usual care, exercise, Western medicine, acupuncture, stimulus control, and placebo music. Regarding PSQI scores, all intervention arms were statistically more effective than usual care, with patients identifying music listening as the most effective intervention (Standardized mean difference or SMD: −0.61, 95% Credible Interval or CrI: −1.01 to −0.20). For overall sleep quality, only music-associated relaxation was statistically superior to usual care (SMD: −0.28, 95% CrI: −0.48 to −0.08). In terms of sleep onset latency, both music-associated relaxation and music listening showed significant benefits (−0.26, −0.64 to −0.09, and −0.28, −0.53 to −0.02); music listening and music combined with exercise tended to enhance sleep efficiency. The authors concluded that music interventions appeared to provide clear benefits for adults with primary insomnia, recommending music listening and music-associated relaxation as the most promising options. In another smaller meta-analysis by de Niet et al. (2009) five randomized controlled trials with six treatment conditions were included, involving 170 participants in the intervention groups and 138 controls. This analysis found that music-assisted relaxation had a moderate impact on improving sleep quality in patients with sleep complaints (SMD, −0.74; 95% CI: −0.96, −0.46).

Despite increasing evidence that listening to music helps improve sleep quality, there is a lack of understanding of the specific types of music or music characteristics that work to improve sleep quality. In past studies, many different types of music interventions have been used for sleep, representing various music genres and artists, music of different tempos, melodies, or structures, music with or without lyrics, music with incorporation of brain waves, particularly delta waves, music familiar to patients, and white noise. In some studies, there was a lack of description of the details of music other than the generic term “relaxation music,” with the characteristics of music taken for granted or overlooked. The ways in which music intervention was delivered also varied widely, with different lengths, frequencies, durations, and passive vs. active interventions. There is also the intrinsic difficulty in blinding patients to music intervention. In this review, we aim to sift through various types of music and music characteristics to identify the elements that make music work to improve sleep quality.

## 2 Methods

### 2.1 Search strategy and databases

This narrative review was informed by a comprehensive literature search of PubMed, MEDLINE, and Google Scholar databases from January 1990 to August 2024. Search terms combined variations of “music,” “sleep,” “insomnia,” “music intervention,” and “music therapy” (e.g., “*music AND sleep,” “insomnia AND music therapy,” “sleep quality AND music intervention”*). Reference lists of key papers were manually screened to identify additional relevant studies.

### 2.2 Eligibility criteria

Studies were included if they:

1. Were randomized controlled trials (RCTs) evaluating music interventions for sleep-related outcomes;
2. Performed in adult population with primary sleep complaints or sleep complaints co-morbid with a medical condition;
3. Reported statistically significant improvements in at least one validated sleep outcome (e.g., PSQI, sleep-onset latency, total sleep time, sleep efficiency); and
4. Provided sufficient information about the music characteristics or delivery methods.

Exclusion criteria included: Non-RCT study design (e.g., observational, quasi-experimental), studies unrelated to sleep outcomes, insufficient methodological or intervention detail, and lack of statistically significant results.

### 2.3 Screening and selection process

**Figure 1:**
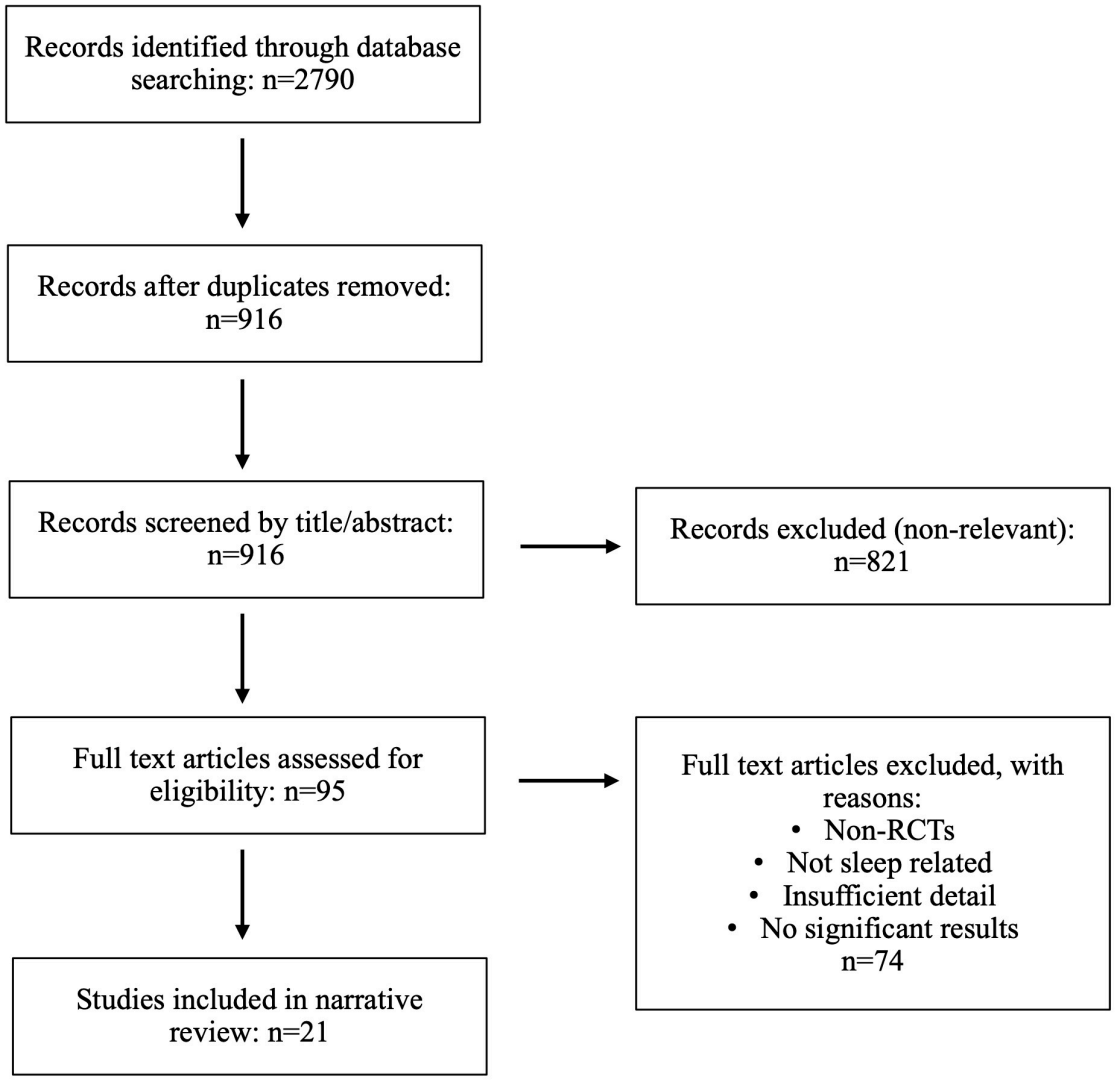
Flow diagram of the study selection process.

The literature search identified 2,790 records. After removing 1,874 duplicates, 916 titles and abstracts were screened for relevance. Ninety-five full-text articles were assessed for eligibility, of which 74 were excluded for not meeting inclusion criteria (non-RCTs, non-sleep focus, insufficient details, or non-significant findings). A total of 21 RCTs were included in this narrative review. The study selection process was illustrated in Figure 1.

### 2.4 Data extraction and synthesis

From each included study, data were extracted on: Music genre and artist, music selection method, intervention duration, frequency, and timing, listening apparatus and environment, participant population characteristics, and reported sleep outcomes. When extracting data, we recorded whether the music was researcher-selected, participant-selected, or co-selected, as shown in Table 1. Because the included studies were methodologically heterogeneous, a qualitative synthesis was conducted rather than a quantitative meta-analysis. Findings were grouped by music genre, musical characteristics, delivery method and environment, and population studied.

**Table 1:** Summary of music genres, artists, instruments, and selectors in reviewed randomized controlled trials.

**Authors**	**Genres**	**Artists**	**Instruments**	**Selected by**
Amiri 2019	Persian: Dastgahs of Nava and Bayat-e Es	Mohammad Reza Shajarian, Parviz Meshkatian, Hossein Alizadeh, Hossein Behroozinia, Ali Pajooheshgar, Masoud Shaari, Mohammad Reza Lotfi, Faramarz Payvar, Alireza Eqekhari, Salar Aghili, Amir Motavalli, and Gholam Hossein Banan	Setar, tar, tonbak, kamancheh, oud and daf	Researchers
Burrai 2020	Western classical	Multiple (80 tracks including 19 pieces from Einaudi, 8 from Bach, 7 from Chopin, 6 from Mozart, 5 from Beethoven, 4 from Debussy, 3 from Pachelbel, Battiato and Liszt, and fewer pieces from multiple additional artists)	Not specified	Researchers
Cai 2015	Chinese music of traditional five elements (including Gong, Shang, Jue, Zhi, and Yu tones)	Not specified	Not specified	Researchers
Chang 2012	Chinese, Taiwanese, Czech, and author-composed	One piece composed by authors. Authors for the other pieces were not specified	Not specified	Participant chose preferred music first, then researcher selected if preferred music was not chosen
Harmat 2008	Western classical (popular pieces from baroque to romantic)	Not specified	Not specified	Researchers
Huang 2017	3 peaceful Buddhist songs or 7 peaceful Buddhist music videos	Not specified	Not specified	Researchers
Jaspersen 2019	Western classical, new age, jazz, and ambient	Not specified	Not specified	Participants chose from 4 playlists selected by researchers
Lai 2005	5 pieces of Western music (New Age, electric, popular oldies, classical, and slow jazz) and 1 piece of Chinese folk music	Not specified	Synthesizer (new age), harp (electric), piano (popular oldies), orchestra (classical), and slow jazz. Orchestra for Chinese folk music	Researchers
Liu 2016	Taiwanese orchestral, Western classical, nature sounds, lullabies, and crystal baby music	Not specified	Not specified	Researchers
Momennasab 2018	New age	Taylor Mesple	Piano	Researchers
Shum 2014	Western classical, Chinese classical, New Age, and jazz	Western classical (Bach: Allemande, Sarabande; Mozart: Romance from Eine Kleine Nachtmusik; Chopin: Nocturne); Chinese classical (Spring River in the Moonlight; Variation on Yang Pass); New Age (Shizuki, Lord of the Wind) and jazz (Everlasting; Winter Wonderland; In Love in Vain)	Not specified	Researchers
Wang 2016	Chinese classic, Western classic, natural sounds music and classical songs without lyrics	A database of 169 pieces of music developed by researcher, a local musician and expert team	Not specified	Participants selected from database developed by researchers
Chan 2010	Meditative, Chinese classical, Western classical, and western modern jazz	Not specified	Not specified	Researchers provided choices to participants
Huang 2016	3 peaceful Buddhist songs or 7 peaceful Buddhist MVs	Not specified	Not specified	Researchers
Lai 2015	7 peaceful Buddhism MVs	Not specified	Not specified	Researchers
Levin 1998	Brain music	Authors	Piano	Researchers
Su 2013	4 pieces of sedating music	Authors	Piano	Researchers
Ziv 2008	Not specified	Not specified	Piano with background violins and bells	Researchers
Bang 2019	Auditory binaural beat (BB): theta BB: 6 Hz shift. Classical, nature sounds or pop songs	Not specified	Not specified	Researchers
Blanaru 2012	Not specified	Not specified	Piano with background violins and bells	Researchers
Hernández-Ruiz 2005	Not specified	Not specified	Not specified	Participants (in 3 cases, participants were unable or unwilling to select music, so researchers selected music in their preferred genre)

### 2.5 Reporting framework

Although this work is a narrative review, we followed the PRISMA 2020 reporting guidelines where applicable to ensure transparency.

## 3 Results

### 3.1 Music genres and artists

Numerous studies have explored the influence of music on sleep quality. This review focuses solely on randomized controlled trials that have shown a statistically significant positive impact of music on participants, as summarized in Table 1. The music interventions in these studies featured a range of genres and artists, with most using instrumental music. Western classical music was the most frequently used genre, appearing in nine of the listed trials (Burrai et al., 2020; Chan et al., 2010; Harmat et al., 2008; Jespersen et al., 2019; Lai and Good, 2005; Liu et al., 2016; Shum et al., 2014; Su et al., 2013; Wang et al., 2016). Some studies specified the artists, whereas others did not. Burrai et al. (2020)'s study offered the most extensive selection of Western classical music, with 80 tracks from various artists available to participants. Among these, 19 tracks were by Einaudi, 8 by Bach, 7 by Chopin, 6 by Mozart, 5 by Beethoven, 4 by Debussy, and 3 each by Pachelbel, Battiato, and Liszt, along with fewer pieces from several other artists. The second most common genre was New Age, featured in five trials (Jespersen et al., 2019; Lai and Good, 2005; Momennasab et al., 2018; Shum et al., 2014; Su et al., 2013). Modern jazz appeared in four studies (Chan et al., 2010; Lai and Good, 2005; Shum et al., 2014; Su et al., 2013). Other less frequently chosen Western music genres included electric (2 studies; Lai and Good, 2005; Su et al., 2013) and popular oldies (2 studies; Lai and Good, 2005; Su et al., 2013).

Another very popular choice of music, especially among Asian researchers, was Asian music. Among the Asian music selected, there was a prevalence of music of Chinese origin, including Chinese classical music which was selected by three research groups (Chan et al., 2010; Shum et al., 2014; Wang et al., 2016), Chinese music based on traditional five elements (including Gong, Shang, Jue, Zhi, and Yu tones) selected by Cai et al. (2015), Chinese folk music selected by Lai and Good (2005), and other Chinese/Taiwanese music selections (Chang et al., 2012; Liu et al., 2016). Religious music of Asian origin was also used in certain studies. Lai et al. (2015) and Huang et al. (2017) chose three peaceful Buddhist songs and seven peaceful Buddhist music videos as their music intervention. The music videos showed nature scenes and captions showing Buddha's teachings.

Another common choice of music intervention was nature sounds, which were included in three of the listed studies (Bang et al., 2019; Liu et al., 2016; Wang et al., 2016). A variety of additional, less commonly employed music types included lullabies (Liu et al., 2016), crystal baby music (Liu et al., 2016), pop songs (Bang et al., 2019), meditative music (Chan et al., 2010), and Czech music (Chan et al., 2010).

The cultural influence on music selection is evident. Just as Asian music was strongly favored in clinical trials carried out by Asian research groups, Persian traditional music of two Dastgahs of Nava and Bayat-e Esfahan were chosen by a research group in Iran, and was played using traditional Persian instruments such as setar, tar, tonbak, kamancheh, oud, and daf (Amiri et al., 2019).

A small number of music selections used in these studies were composed by the authors or researchers themselves. For example, in a study on the effects of music on the sleep quality of adults with chronic insomnia, Chang et al. (2012) composed a piece of sedating music. Similarly, four pieces of sedating music were composed by the authors and used in their study of the effect of music on sleep quality in patients in the medical ICU by Su et al. (2013).

It is also noted that in a few studies, personal preferred music was selected by participants (Chang et al., 2012; Hernández-Ruiz, 2005; Wang et al., 2016). In these cases, details about the music genre or artists were typically not provided, and it was often assumed that music familiar to the participants would be more helpful in improving sleep, although evidence supporting this assumption remains to be investigated. It should also be noted that familiarity is a dynamic state that can be gained by repeated exposure to specific pieces.

### 3.2 Characteristics of music used in studies

Although a diverse array of music genres and artists were found in the studies reviewed, these studies consistently utilized music with specific characteristics to promote relaxation and improve the sleep quality. The predominant feature was a slow tempo, typically ranging from 60 to 80 beats per min (bpm), which aligns with the average resting heart rate. This tempo is believed to facilitate relaxation by mimicking the body's natural rhythms (Burrai et al., 2020; Nilsson, 2009). Other common musical elements include minor tonalities, soft and smooth melodies, and simple structures (Blanaru et al., 2012; Chan et al., 2010; Chang et al., 2012; Huang et al., 2017, 2016; Jespersen et al., 2019; Lai et al., 2015; Su et al., 2013; Wang et al., 2016; Ziv et al., 2008).

These musical characteristics align with Gaston's definition of sedative music, which is designed to induce relaxation in listeners (Gaston, 1951). For instance, Shum et al. (2014) employed soft, instrumental, slow music without lyrics, played at 60–80 bpm for 40 min. Some studies, such as the 1998 Levin study, incorporated additional elements, such as legato and staccato (Levin YaI, 1998). Conversely, certain features were deliberately avoided in music interventions, including lyrics, accented beats, percussive characteristics, and syncopation (Chang et al., 2012; Lai et al., 2015; Shum et al., 2014). This careful selection of musical elements demonstrates a targeted approach to creating an auditory environment that is conducive to relaxation and improved sleep.

### 3.3 Brain music and other alternative music forms

Inspired by the resemblance between brain waves and musical rhythms, numerous scientists have explored potential links between the two. Various translation methods have been employed to transform brain waves into music, predominantly relying on the spectral characteristics of EEGs. In a pioneering study conducted by Levin in 1998, researchers devised a unique algorithm to convert 16-channel EEG recordings into music that represents four stages of sleep waves, which they termed “brain music” (Levin YaI, 1998). This randomized controlled trial revealed that brain music had beneficial effects on over 80% of patients with insomnia, as assessed using both subjective and objective criteria.

In a separate study, Wu et al. (2009) created scale-free brainwave music by applying a series of rules. These included directly mapping the period of an EEG waveform to the length of a musical note, using a logarithmic mapping of the change in average EEG power to music intensity based on Fechner's law, and employing a scale-free mapping from EEG amplitude to music pitch according to the power law. The researchers applied these rules to EEG segments recorded during rapid eye movement (REM) sleep and slow-wave sleep (SWS). Sixty volunteers assessed 25 music pieces, with 10 from the REM group, 10 from the SWS group, and 5 from white noise (WN). Among the participants, 74.3% reported feeling happy when listening to REM music and found SWS music boring and sleep-inducing, with an average identification accuracy of 86.8% (*k* = 0.800; *P* < 0.001). The same music generation rules were used in a more recent study (Gao et al., 2020), where 33 young participants were randomly assigned to listen to REM brain wave music, SWS brain wave music, or a white noise (WN) control group for 20 min before bedtime over 6 days. The study revealed improved sleep efficiency in the SWS group, while the REM and WN groups experienced decreased efficiency, indicating that SWS brainwave music might have a particularly beneficial impact on sleep quality.

A recent randomized controlled trial explored the effects of binaural beats (BB) on sleep quality (Bang et al., 2019). Auditory BB is subjectively perceived as an auditory beat that equals the difference between two similar tones delivered to each ear separately. For instance, if a 200-Hz tone is played in the left ear and a 220-Hz tone in the right ear, a 20 Hz beat is not directly heard but is processed by the brain. When a specific BB is maintained, it can lead to brainwave entrainment. However, the study found that using auditory BB with a beat frequency akin to theta brain waves did not significantly enhance sleep quality compared to using pure music. It did, however, have the potential to modify brain activity, increasing daytime alertness in individuals with subclinical insomnia. A notable limitation of this study was the requirement for specialized audio equipment capable of inducing target brain waves through BB.

### 3.4 Music intervention dosing and delivery

In the studies reviewed, various dosing and delivery methods for music intervention were employed, as detailed in Table 2. Regarding the duration of music intervention sessions, most researchers opted for sessions lasting between 30 and 45 min, with 30-min sessions being the most common choice (Bang et al., 2019; Burrai et al., 2020; Cai et al., 2015; Chan et al., 2010; Huang et al., 2017, 2016; Jespersen et al., 2019; Lai et al., 2015; Liu et al., 2016). This was followed by 45-min sessions (Chang et al., 2012; Harmat et al., 2008; Lai and Good, 2005; Ziv et al., 2008) and 40-min sessions (Blanaru et al., 2012; Shum et al., 2014). In the study conducted by Wang et al., the sessions ranged from 30 to 45 min. The longest session, lasting 60 min, was reported by Amiri et al. (2019). Another study (Momennasab et al., 2018) involved relatively long 50-min sessions for hemodialysis patients. The shortest session was found in Levin's research, which utilized 13-min-long author-composed “brain music” for treating insomnia. Shorter 20-min sessions were used to examine sleep patterns among abused women residing in shelters (Hernández-Ruiz, 2005). Most studies implemented a daily music intervention, except for a crossover controlled trial in which the intervention was administered for two consecutive days each week, alternating with brisk evening walks (Huang et al., 2016).

**Table 2:** Summary of music intervention dosing, delivery, participants, and study environments.

**Authors**	**Length**	**Frequency**	**Duration**	**Listening apparatus**	**Participants**	**Study environments/location**
Amiri 2019	60 min	Daily between 10 and 11 p.m.	6 weeks (42 days)	Not reported	30 men with primary insomnia (mean age 27 ± 2.5)	Participants' homes in Iran
Burrai 2020	At least 30 min/day	Daily, anytime of the day	90 days	MP3 player with headphone	159 patients with heart failure (99 men, 60 women, mean age 71.6 ± 12 for experiment group and 74.6 ± 10.9 for control)	Homes of participants who were patients from 4 cardiology centers in Italy
Cai 2015	30 min	Daily (did not specify what time of the day)	30 days	Not reported	154 patients with post-stroke insomnia (83 men, 71 women, mean age 63.9 ± 10.4 for music group and 64.5 ± 12.6 for control)	A rehabilitation clinic in China
Chang 2012	45 min	Daily at bedtime	3 days	CD player with speakers	50 adults with chronic insomnia (3 men, 47 women, mean age 32 ± 11, range 22–58)	A sleep lab in a hospital in Taiwan
Harmat 2008	45 min	Daily, bedtime	3 weeks	Not reported	94 students with sleep complaints (21 men, 73 women, mean age 22.6 ± 2.9, range 19–28)	Participants' homes in Hungary
Huang 2017	30 min	Daily. Songs: 4 days in a row before bedtime. MVs: at least 2 h before bedtime	6 days	Mobile phones with or without earphones	48 adults with sleep disturbances (9 men, 39 women, mean age 41 ± 16.7, range 22–67)	Patients recruited from the outpatient department of a hospital in Taiwan. 1-channel EEG machine set up at home
Jaspersen 2019	30 min	Daily, bedtime	21 days	Audio player with speakers	57 participants with insomnia (12 men, 45 women, mean age 50.2 ± 11.6, range 18–65)	Homes of patients from Denmark, UK, and Netherlands
Lai 2005	45 min	Daily, bedtime	3 weeks	Audio tape player with or without earphones	60 older adults with sleeping difficulty (mean age 67 ± 5, range 60–83)	Community centers in Taiwan
Liu 2016	30 min	Daily, bedtime	14 days	Not reported	121 pregnant Taiwanese women with poor sleep quality (age not reported)	Participants' homes in Taiwan
Momennasab 2018	50 min	Daily, during dialysis and at bedtime	4 weeks	Not reported	102 dialysis patients (56 men, 46 women, mean age 49.86 ± 11.12, range 18–60)	A dialysis center in southern Iran
Shum 2014	40 min	Daily (did not specify what time of the day)	5 weeks	MP4 player with earphones	60 community dwelling adults with poor sleep quality (PSQI scores >5) (20 men, 40 women, mean age 64, range 57–68)	Participants' homes in one community center in Singapore
Wang 2016	30–45 min	Daily, bedtime	3 months	MP3 player with earphones	64 older adults aged >60 with poor sleep quality (13 men, 51 women, mean age 69 ± 5.46)	Participants' homes in four urban community centers in China
Chan 2010	30 min	Daily (did not specify what time of the day)	4 weeks	MP3 player with earphones	42 elderly people aged >60 (19 men, 23 women, majority aged 75 or above)	Participants' homes in one community center in Hong Kong
Huang 2016	30 min	2 consecutive days per week before bedtime, alternating with music + walking in another week	2 weeks	Not reported	38 community residing people aged 50–75 with insomnia (8 men and 30 women, mean age 56.42 ± 7.35)	Participants' homes at community centers in Taiwan
Lai 2015	30 min,	2 consecutive nights at bedtime	2 nights	TV	38 subjects with chronic insomnia aged 50–75	A sleep center in a hospital in Eastern Taiwan
Levin 1998	13 min	Daily, bedtime	15 days	Not reported	58 patients with insomnia aged 18–60 (60.3% women, mean age 43)	A sleep lab in Russia
Su 2013	45 min	1 night	1 night	CD player with speakers	28 ICU patients aged 39–78 (17 men and 11 women, mean age 61.68 ± 9.82)	A medical ICU in a Taipei, Taiwan hospital
Ziv 2008	40 min	Daily (did not specify time of the day)	1 week	Not reported	15 patients aged 67–93 with insomnia (4 men and 11 women, mean age 80.63 ± 6.85)	Participants' homes in community housing in Israel
Bang 2019	30 min	Daily, bedtime	2 weeks	Binaural beat audio apparatus with wireless headset	43 patients with subclinical insomnia (11 men, 32 women, mean age 34.3 ± 10.4, range 20–59)	Participants' homes in Gyeonggi-do, South Korea
Blanaru 2012	40 min	Daily, bedtime	2 weeks	Not reported	13 psychiatric clinic patients with PTSD (8 men, 5 women, mean age 45.7 ± 11.4)	Participants' homes in Afula, Israel
Hernández -Ruiz 2005	20 min	Daily, during the day	5 days	Portable CD player	28 abused women in shelters (mean age 35.36)	2 domestic violence shelters in a US midwestern city

In most studies, music interventions were delivered to participants before bedtime. An exception was a study on classical music in patients with heart failure, in which patients were allowed to listen to music at any time of the day (Burrai et al., 2020). Additionally, in a study involving abused women in domestic violence shelters, music intervention was delivered during interviews with researchers during the day (Hernández-Ruiz, 2005).

The length of the music interventions varied significantly, with most studies lasting between 1 and 4 weeks. The most frequently used duration was 2 weeks, as seen in four studies included in this review (Bang et al., 2019; Blanaru et al., 2012; Huang et al., 2016; Liu et al., 2016). Following this, three-week sessions were implemented in three studies (Harmat et al., 2008; Jespersen et al., 2019; Lai and Good, 2005). Some studies opted for shorter durations of less than a week. For instance, Su et al. (2013) found that a single night of music intervention improved sleep quality in medical ICU patients. Similarly, Lai et al. (2015) observed positive effects on sleep quality in individuals aged 50–75 with chronic insomnia after two consecutive nights of music. Other studies with brief durations included 3-day sessions by Chang et al. (2012), 4-day sessions by Momennasab et al. (2018), and 5-day sessions by Hernández-Ruiz (2005). In contrast, the studies by Burrai et al. (2020) and Wang et al. (2016) extended over 3 months. Longer durations were also seen in the Amiri et al. (2019) study, which lasted 6 weeks, and the Shum et al. (2014) study, which spanned 5 weeks.

Although the majority of studies did not detail the precise volume at which music was played, those that did indicated a comfortable level. In the study by Burrai et al. (2020) the music volume was set between 50 and 60 dB, significantly lower than the 85 dB safety limit for portable media devices such as MP3 players. Wang et al. (2016) directed their participants to listen to music at a comfortable volume (60 dB) or the lowest audible level using earphones. In the study by Huang et al. (2016) participants adjusted the music volume to a level they found comfortable.

A significant number of studies did not disclose the specific listening devices employed. For those that did provide this information, the devices varied and included CD players, MP3/MP4 players, mobile phones, and televisions. The sound was delivered through either built-in speakers or earphones. Notably, the research conducted by Bang et al. (2019) employed a specialized audio apparatus capable of generating binaural beats, which was paired with a wireless headset.

### 3.5 Music intervention environments and patient populations

The studies reviewed encompassed a range of environments for music interventions. The majority took place in participants' homes, including community center residences across different countries and regions (Bang et al., 2019; Burrai et al., 2020; Chan et al., 2010; Harmat et al., 2008; Huang et al., 2017; Jespersen et al., 2019; Lai and Good, 2005; Levin YaI, 1998; Liu et al., 2016; Shum et al., 2014; Wang et al., 2016; Ziv et al., 2008). Two studies conducted music interventions in hospital sleep laboratories (Huang et al., 2016; Lai et al., 2015). Additional settings included a dialysis center in southern Iran (Momennasab et al., 2018), a rehabilitation clinic in China (Cai et al., 2015), a medical ICU in a hospital in Taiwan (Su et al., 2013), and two domestic violence shelters in a midwestern US city (Hernández-Ruiz, 2005).

In terms of the patient population studied, most studies included adult participants with chronic insomnia or various sleep difficulties. However, very diverse participant pools were included in several of the studies. For example, 121 pregnant women with poor sleep quality were enrolled in Liu et al.'s study (2016). Other populations studied included 150 dialysis patients in southern Iran (Momennasab et al., 2018), 28 ICU patients aged 9–78 in a hospital in Taiwan (Su et al., 2013), 13 PTSD patients with a mean age of 45.7 years in Israel (Blanaru et al., 2012), 159 patients with heart failure in Italy (Burrai et al., 2020), and 28 abused women in domestic violence shelters in the US (Hernández-Ruiz, 2005).

## 4 Discussion

While our review supported the notion that music from a wide variety of genres and artists could help people improve their sleep, classical music that is prevalent in the participants' cultural environment and music that is familiar to the participants may be more favorable. Specific genres worth mentioning include Western classical and New Age. For those who are religious, a selection of soothing religious music could result in greater benefits. Nature sounds seem to be another good option for improving sleep quality. Alternative music forms such as brain music or brain wave music also hold promise for improving sleep quality, although more updated and large-scale randomized controlled trials are needed to further validate their therapeutic efficacy.

The following characteristics of music were shown to improve sleep quality: slow tempo of 60–80 bpm, minor tonalities, soft and smooth melodies, and simple structure. The characteristics of music that enhance sleep quality are rooted in their ability to induce relaxation and reduce physiological arousal. A slow tempo of 60–80 beats per min mimics the resting heart rate, promoting a sense of calmness and facilitating the transition to sleep (Burrai et al., 2020; Nilsson, 2009). Minor tonalities evoke a soothing atmosphere, whereas soft and smooth melodies create a gentle auditory environment that does not stimulate alertness. The simple structure of such compositions prevents cognitive engagement, allowing the mind to gradually disengage from active thought processes.

When composing music specifically to improve sleep quality, it is important to avoid elements that may stimulate or engage the listener. The features include lyrics, accented beats, percussive characteristics, and syncopations. Lyrics can activate language processing centers in the brain, potentially keeping the mind active. Accented beats and percussive characteristics can create sudden auditory stimuli that may startle or alert listeners. Syncopation, with its unexpected rhythmic patterns, may require more cognitive processing and attention than other musical elements. By carefully considering these factors and incorporating beneficial elements while avoiding potentially disruptive features, composers can create music that effectively supports the sleep process and enhances overall sleep quality.

Recent survey-based studies of habitual music-for-sleep users (Dickson and Schubert, 2019; Scarratt et al., 2023; Trahan et al., 2018) revealed wide variation in musical choices, including popular and lyrical songs. In an online survey conducted with 651 members of the general public, Trahan et al. (2018) discovered that classical music is the most prevalent genre, with Bach, Mozart, and Chopin ranking among the top artists, aligning with the findings of this review. However, a wider range of musical preferences was noted, encompassing 14 different genres and 545 artists in total. Scarratt et al. (2023) analyzed an extensive global dataset comprising 225,626 tracks from the music streaming platform Spotify and discovered even more diversity in the selection of sleep music. Their findings indicated that sleep music is generally softer, slower, and more often instrumental (i.e., without lyrics). However, classical music only ranked as the 7th most common genre. Surprisingly, three of the six subgroups categorized in their study contained popular tracks that were faster, louder, and more energetic than average sleep music. These findings contrast with experimental literature that predominantly employs instrumental or classical music. The discrepancy likely reflects differences between controlled study conditions and real-world usage. Incorporating participant-chosen or ecologically valid music into future trials may yield intriguing and more generalizable results.”

While many studies used 30–45 min sessions before bedtime, few directly compared different intervention durations. Therefore, this timeframe should be regarded as common practice rather than an empirically established optimum. This duration allows sufficient time for the body and mind to respond to the soothing effects of music and gradually transition into a relaxed state conducive to sleep. However, flexibility in duration is important, as individual needs may vary based on factors such as stress levels, personal preferences, and daily routines. Some individuals may benefit from longer sessions, whereas others might find shorter periods equally effective.

The environment and manner in which music is delivered play significant roles in maximizing therapeutic potential. A comfortable setting free from distractions enhances the listener's ability to focus on and absorb music. The volume should be kept low, creating a gentle auditory backdrop rather than an overwhelming sensory experience. This approach allows music to subtly influence physiological processes, such as heart rate and breathing, without causing stimulation that could counteract its calming effects. The type of listening apparatus used is also an important consideration, which was not reported in many studies. Downs (2021) proposed that using earphones or headphones might enhance a feeling of personal space and comfort, which could aid sleep, though it might also present practical challenges, such as issues with physical comfort. By carefully considering these factors—timing, duration, volume, and environment—individuals can optimize the positive impact of music intervention on their sleep quality and overall wellbeing.

Most studies employed researcher-selected music without detailing the rationale or pre-testing procedures. This introduces potential bias, as researcher assumptions about what is “relaxing” may not align with participant preference or cultural background. While some trials allowed limited participant choice (e.g., Chang et al., 2012; Wang et al., 2016), the absence of standardized selection methods complicates interpretation of efficacy. Future research should report the basis for music choice and consider preference-adaptive designs.

This review was limited to randomized controlled trials that demonstrated statistically significant improvements in sleep, potentially leading to an overrepresentation of positive outcomes while omitting studies with no observed effects. For example, a few studies, including those by Lazic and Ogilvie (2007) and Koenig et al. (2013), reported no significant results when using music (Koenig et al., 2013; Lazic and Ogilvie, 2007). In Lazic and Ogilvie (2007) research, Thompson's Delta Sleep System was utilized. Although Thompson promotes the Delta Sleep System as enhancing delta brain-wave activity and improving sleep, we did not find any published peer-reviewed randomized controlled trial supporting this claim. Koenig et al. used relaxing classical music, which shares similar structural characteristics with many trials reviewed here. However, both studies involved healthy young adult undergraduate college students without sleep problems, which was not the target population for this review. Future reviews should systematically compare both effective and ineffective interventions to delineate the boundary conditions under which music facilitates sleep.

We recognize that our review has several additional limitations. First, we did not explore the mechanisms by which music enhances sleep. One possible explanation is that music may lower cortisol levels, thereby reducing stress, activating the parasympathetic nervous system, and influencing physiological factors such as blood pressure, heart rate, and respiratory rate (Nilsson, 2009, 2008). Nonetheless, the precise mechanisms through which music affects sleep are likely to be complex and multifaceted, which is beyond the scope of this review. Another limitation is that the majority of the studies we examined focused on adults, leaving the impact of music interventions on younger populations underexplored. Additionally, all the studies reviewed had a duration of 3 months or less, raising questions about the long-term benefits of music therapy.

In the future, further research on the effects of music on sleep will be crucial. A comparison of compositions using the above-described positive characteristics with commercial music available on the internet, often with extremely high view counts, using a randomized controlled study, could shed light on the effectiveness of popular music-based sleep remedies and provide insights into the improvements of sleep music, potentially benefiting a large audience. With the advent of artificial intelligence, creating music that incorporates the features discussed in this review will be greatly expedited. Longer-duration studies on the effects of music therapy will also offer more insights into the long-term benefits of music on sleep quality. Studies targeting specific populations not covered in the reviewed trials, such as the youth population, will also be beneficial in advancing our knowledge. Furthermore, due to the variety of methodologies employed, the incomplete reporting of methodological specifics, and the overall lack of uniformity among studies, we suggest that future research should: (1) report music selection methods and rationale; (2) describe playback apparatus, volume settings, and intervention environment; (3) test participant-selected vs. researcher-selected music; (4) compare session lengths and timing; (5) report null outcomes to reduce publication bias; and (6) use preregistered protocols. Larger randomized and preference-adaptive trials are essential to identify reliable parameters for sleep-promoting music.
